# Sirtuin Oxidative Post-translational Modifications

**DOI:** 10.3389/fphys.2021.763417

**Published:** 2021-11-24

**Authors:** Kelsey S. Kalous, Sarah L. Wynia-Smith, Brian C. Smith

**Affiliations:** Department of Biochemistry, Medical College of Wisconsin, Milwaukee, WI, United States

**Keywords:** sirtuin (SIRT), nitrosation, glutathionylation, sulfhydration, sulfenylation, nitration, nitrosylation, oxidation

## Abstract

Increased sirtuin deacylase activity is correlated with increased lifespan and healthspan in eukaryotes. Conversely, decreased sirtuin deacylase activity is correlated with increased susceptibility to aging-related diseases. However, the mechanisms leading to decreased sirtuin activity during aging are poorly understood. Recent work has shown that oxidative post-translational modification by reactive oxygen (ROS) or nitrogen (RNS) species results in inhibition of sirtuin deacylase activity through cysteine nitrosation, glutathionylation, sulfenylation, and sulfhydration as well as tyrosine nitration. The prevalence of ROS/RNS (e.g., nitric oxide, *S*-nitrosoglutathione, hydrogen peroxide, oxidized glutathione, and peroxynitrite) is increased during inflammation and as a result of electron transport chain dysfunction. With age, cellular production of ROS/RNS increases; thus, cellular oxidants may serve as a causal link between loss of sirtuin activity and aging-related disease development. Therefore, the prevention of inhibitory oxidative modification may represent a novel means to increase sirtuin activity during aging. In this review, we explore the role of cellular oxidants in inhibiting individual sirtuin human isoform deacylase activity and clarify the relevance of ROS/RNS as regulatory molecules of sirtuin deacylase activity in the context of health and disease.

## Introduction to Sirtuins

Sirtuins are a class of enzymes that remove acetyl and longer-chain acyl groups from lysine residues on proteins in an NAD^+^-dependent manner, producing *O*-acyl-ADP-ribose, nicotinamide, and deacylated lysine as products (Feldman et al., 2012). Humans encode seven sirtuin isoforms (Sirt1–7) with distinct subcellular distribution (Table 1; Michishita et al., 2005; Houtkooper et al., 2012). Sirt1 and Sirt2 shuttle between the nucleus and cytoplasm (North and Verdin, 2007; Tanno et al., 2007). Sirt3 (Onyango et al., 2002; Schwer et al., 2002; Michishita et al., 2005; Hallows et al., 2008) and Sirt4 (Michishita et al., 2005; Ahuja et al., 2007) are primarily localized to the mitochondria. Sirt5 is primarily mitochondrial (Michishita et al., 2005; Carrico et al., 2018), although Sirt5 also regulates the acylation levels of cytosolic targets (Park et al., 2013; Nishida et al., 2015). Sirt6 and Sirt7 reside in the nucleus (Michishita et al., 2005), with Sirt7 further localized to the nucleolus (Michishita et al., 2005; Chen et al., 2013).

**TABLE 1:** Human sirtuin isoform molecular weight, subcellular localization, and known lysine deacylase targets.

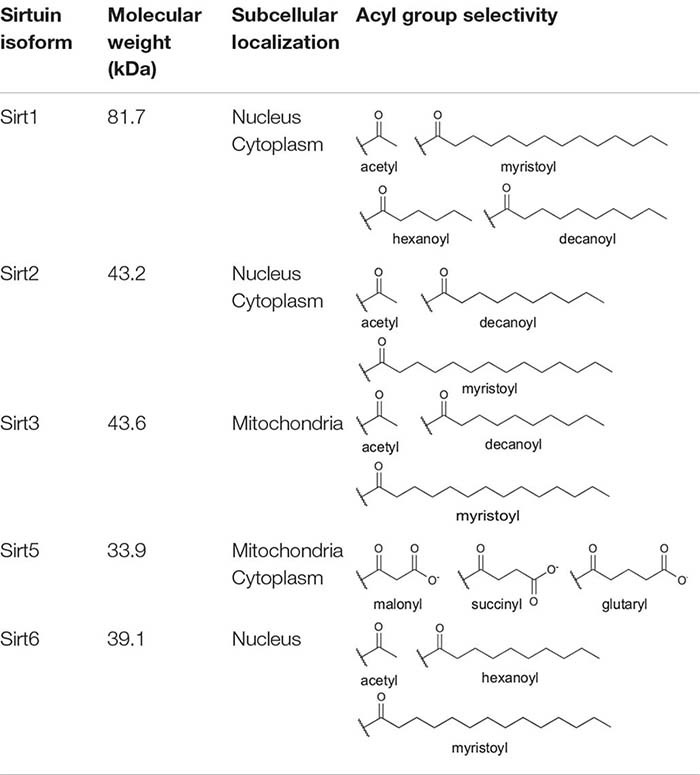

**FIGURE 1:**
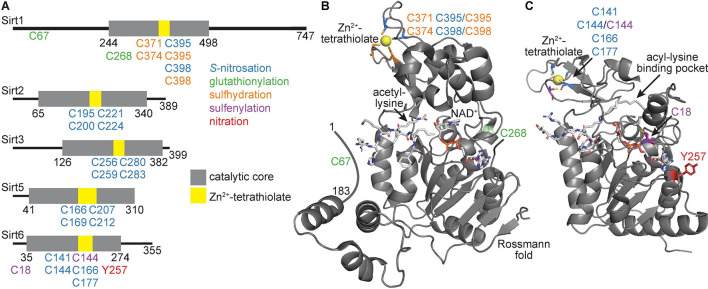
Human sirtuin domain organization and tertiary structure of the catalytic core. **(A)** Linear representation of Sirt1, Sirt2, Sirt3, Sirt5, and Sirt6. Gray represents the catalytic core, with yellow denoting the Zn^2+^-tetrathiolate region. **(B)** Tertiary structure of the catalytic core and activator-binding domain of Sirt1 (PDB ID: 4ZZJ) with structural features and target residues labeled. **(C)** Tertiary structure of the catalytic core of Sirt6 (PDB ID: 7CL1) with structural features and target residues labeled. Blue residues denote sites of *S*-nitrosation, green residues denote sites of glutathionylation, orange residues denote sites of sulfhydration, purple residues denote sites of sulfenylation, and red residues denote sites of tyrosine nitration.

Sirtuin family members display distinct deacylase activities (Table 1; Bheda et al., 2015) and protein targets (Nakagawa et al., 2009) despite a high degree of conservation across isoforms in their sequence and structure (Yuan and Marmorstein, 2012; Bheda et al., 2015). Indeed, all seven sirtuins contain a core catalytic domain harboring a four-coordinate cysteine Zn^2+^ finger (the Zn^2+^-tetrathiolate subdomain) with a structurally adjacent acyl-lysine binding pocket (Figure 1). Likewise, each sirtuin has an antiparallel β-sheet-rich Rossmann-fold subdomain that binds NAD^+^.

Sirtuin deacylase activity is important for health and survival, both at the cellular and organismal level; increased sirtuin activity is generally correlated with increased lifespan and healthspan (Imai and Guarente, 2016). Decreased sirtuin activity is generally correlated with development of aging-related diseases including cardiovascular disease (Sebastián et al., 2012), type II diabetes (Guarente, 2010; Haigis and Sinclair, 2010), neurodegeneration (Ansari et al., 2017), and cancer (Alhazzazi et al., 2011). Human sirtuins have far too many reported acylated protein substrates to comprehensively cover in this review; thus we focus on a subset of known deacylase substrates involved in metabolism, redox homeostasis, or inflammation (Table 2) as well as those used to show cellular sirtuin inhibition as discussed below. Although the molecular mechanisms negatively regulating sirtuin activity in aging-related pathologies are not fully understood, mounting evidence indicates (patho)physiological reactive oxygen and nitrogen species (ROS and RNS, respectively) play a role (Merksamer et al., 2013; Santos et al., 2016; Shahgaldi and Kahmini, 2021). ROS/RNS are produced in increasing concentrations with age (Harman, 1956; Finkel and Holbrook, 2000) and may negatively regulate sirtuin activity *via* post-translational modification of critical cysteine and tyrosine side chains. In this review, we cover oxidative post-translational modifications of Sirt1, Sirt2, Sirt3, Sirt5, and Sirt6; we omit discussion of Sirt4 and Sirt7 as they have no known oxidative modifications.

**TABLE 2:** Role of Sirt1, Sirt2, Sirt3, Sirt5, and Sirt6 in maintaining cellular metabolic processes, oxidant homeostasis, and reduction of inflammation.

**Sirtuin isoform**	**Deacylase target**	**Deacylated signaling output**	**References**
Sirt1	p53	Reduced p53 transcription factor activity and apoptosis	Vaziri et al., 2001
	PGC1α	PCG1α activation and induction of gluconeogenic gene expression and hepatic glucose output	Rodgers et al., 2005, 2008
	FOXO1	Repressed FOXO1 transcriptional activation	Motta et al., 2004; Yang et al., 2005
	SREBP1c	Decreased SREBP1c stability and occupancy at lipogenic genes	Ponugoti et al., 2010
	SREBP2	Downregulated SREBP2 target gene expression (e.g., LDL receptor)	Walker et al., 2010
	STAT3	Suppressed repression of liver gluconeogenesis	Nie et al., 2009
	NF-κB	Inhibition of NF-κB transcriptional activity and promotion of TNFα-induced apoptosis	Yeung et al., 2004
	HMGB1	Prevention of HMGB1 cytosolic release and inflammatory activation	Hwang et al., 2014; Rabadi et al., 2015
	tau	Enhanced degradation of phosphorylated tau and prevention of tau aggregates	Min et al., 2010

Sirt2	α-Tubulin	Maintained stability of peritubular microtubule network	North et al., 2003; Skoge and Ziegler, 2016
	CDC20	Regulation of anaphase-promoting complex	Kim et al., 2011
	PEPCK	Stabilized PEPCK and regulation of glucose homeostasis	Jiang et al., 2011
	G6PD	Maintenance of cellular NADPH homeostasis	Wang et al., 2014; Xu et al., 2016
	FOXO1	Negative regulation of FOXO1-dependent autophagy	Zhao et al., 2010
	p65	Deacetylation and inhibition of p65-dependent transcription in response to TNFα	Rothgiesser et al., 2010

Sirt3	OPA1	Maintenance of OPA1 function and associated mitochondrial structural integrity	Samant et al., 2014
	IDH2	Preservation of IDH2 enzymatic activity and proper mitochondrial redox balance	Someya et al., 2010; Yu et al., 2012
	ATPβ	Regulation of mitochondrial ATP balance	Zhang et al., 2016
	SOD2	Increased mitochondrial superoxide detoxification	Qiu et al., 2010; Tao et al., 2014;
			Zhang et al., 2016; Dikalova et al., 2017

Sirt5	CPS1	Increased CPS1 urea cycle activity	Du et al., 2011; Tan et al., 2014
	SHMT2	Activated to drive serine catabolism	Yang et al., 2017
	HMGCS2	Maintenance of ketogenesis	Rardin et al., 2013
	SOD1	Elimination of mitochondrial reactive oxygen species	Lin et al., 2013

Sirt6	H3K9	Modulation of telomeres and repressed NF-κB dependent transcriptional activation	Michishita et al., 2008; Kawahara et al., 2009
	H3K56	Dynamic regulation of telomeric chromatin	Michishita et al., 2009

### Sirt1

Sirt1 is the largest and most studied of the seven human sirtuin isoforms. Although Sirt1 can shuttle between the nucleus and cytoplasm, Sirt1 localization is primarily nuclear (Table 1; Tanno et al., 2007). Sirt1 displays strong deacetylase activity (Feldman et al., 2015). Sirt1 regulates gene transcription *via* deacetylation of transcription factors (Motta et al., 2004; Bouras et al., 2005), transcriptional coregulators (Rodgers et al., 2005, 2008; Cantó et al., 2009, 2010), and other critical cellular signaling proteins including the acetylation-dependent activity of multiple enzymes. Sirt1 is a modulator of apoptosis (Vaziri et al., 2001; Motta et al., 2004; Hughes et al., 2011; Mao B. et al., 2011), inflammation (Yeung et al., 2004), cellular energy status (Pedersen et al., 2007; Ponugoti et al., 2010; Hirschey et al., 2011; Anderson et al., 2017), and DNA repair (Cohen et al., 2004; Jeong et al., 2007; Zhang et al., 2015).

Sirt1 deacetylase activity is at the helm of many metabolic, signaling, and transcription-regulating processes (Table 2). Sirt1 deacetylates p53 in the cytosol, blocking p53 nuclear translocation and ability to induce apoptosis (Vaziri et al., 2001). Likewise, the transcriptional co-regulator peroxisome proliferator-activated receptor-γ co-activator 1α (PGC1α) is a deacetylase target of Sirt1; deacetylation leads to PGC1α activation and induction of mitochondrial gene expression programs (Rodgers et al., 2005, 2008; Cantó et al., 2009, 2010). Sirt1 deacetylates the transcription factor forkhead box protein O1 (FOXO1) (Motta et al., 2004; Yang et al., 2005; Hughes et al., 2011), thereby promoting FOXO1 nuclear retention and transcriptional activation of FOXO1-dependent genes. Sirt1 is tied to lipid metabolism through deacetylation of sterol regulatory element binding proteins 1 and 2 (SREBP1 and SREBP2) (Ponugoti et al., 2010; Walker et al., 2010). Liver metabolism is also affected by Sirt1; repression of hepatic gluconeogenesis by STAT3 is suppressed by Sirt1 deacetylation of STAT3 (Nie et al., 2009; Sestito et al., 2011).

Sirt1 may also serve as a sensor of cellular redox status *via* its deacetylase targets (Table 2). One such target is nuclear factor kappa-light-chain-enhancer of activated B cells (NF-κB); deacetylation of the p65 subunit of NF-κB inhibits NF-κB transcriptional activity and promotes tumor necrosis factor alpha (TNF-α)-induced apoptosis (Yeung et al., 2004). An additional Sirt1 deacetylase target is the redox-sensitive protein high mobility group protein B1 (HMGB1) (Hwang et al., 2014; Rabadi et al., 2015). HMGB1 functions as an alarmin to induce inflammation; when acetylated, HMGB1 is released into the extracellular space (Bonaldi et al., 2003). Sirt1 deacetylates HMGB1, thus preventing its extracellular release and opposing inflammatory activation.

Sirt1 deacetylase activity also has proven important in the nervous system (Table 2). Acetylation of tau, an adapter protein that binds and stabilizes microtubules, disrupts the ability of tau to bind microtubules and promotes tau aggregation. Plaques of hyperacetylated tau are frequently found in the postmortem brains of Alzheimer’s patients (Irwin et al., 2012). Sirt1 interacts with and deacetylates tau in mouse models, preventing tau aggregation (Min et al., 2010).

### Sirt2

Similar to Sirt1, Sirt2 displays cell stimulus-dependent nuclear-cytoplasmic shuttling (Table 1; North and Verdin, 2007). Also similar to Sirt1, Sirt2 is a strong deacetylase but can also deacylate longer acyl chains such as myristoyl (Table 1; Feldman et al., 2015; Teng et al., 2015; Wang Y. et al., 2017; Huang et al., 2018). However, unlike Sirt1 which primarily resides in the nucleus, Sirt2 harbors a nuclear export sequence and is primarily cytosolic (North and Verdin, 2007). The principal known functions of Sirt2 are regulation of cell division (North et al., 2003; Kim et al., 2011) and initiation of inflammatory responses (Table 2; Rothgiesser et al., 2010). In particular, Sirt2 can deacetylate microtubule proteins, including α-tubulin (North et al., 2003), and the cell cycle checkpoint protein CDC20 (Kim et al., 2011), to promote cell division.

In addition to regulation of cell cycle, Sirt2 regulates glucose homeostasis by deacetylation and stabilization of phosphoenolpyruvate carboxykinase (PEPCK) (Jiang et al., 2011) under conditions of nutrient deprivation. Sirt2 also deacetylates glucose-6-phosphate dehydrogenase (G6PD) (Wang et al., 2014; Xu et al., 2016), the rate-limiting enzyme in the pentose phosphate pathway, which activates production of ribose-5-phosphate for nucleotide synthesis and promotes production of the reducing equivalent NADPH in the cytosol to counteract oxidant stress. Similar to Sirt1, Sirt2 deacetylates FOXO1 in response to oxidative stress, which negatively regulates FOXO1-dependent autophagy (Zhao et al., 2010). Like Sirt1, Sirt2 deacetylates and inhibits p65-dependent transcription in response to TNFα (Rothgiesser et al., 2010).

### Sirt3

Sirt3 harbors strong deacetylase activity (Onyango et al., 2002; Schwer et al., 2002) and is primarily restricted to the mitochondria (Table 1; Onyango et al., 2002; Schwer et al., 2002; Michishita et al., 2005; Hallows et al., 2008); therefore, Sirt3 activity plays a critical role in regulation of the mitochondrial acetylome. Sirt3 activity directly regulates both cellular energy metabolism and oxidative stress burden *via* deacetylation and subsequent activation of key metabolic and oxidant detoxification enzymes (Table 2). Under stress conditions, the mitochondrial fusion-involved protein optic atrophy 1 (OPA1) becomes hyperacetylated. Sirt3 deacetylation of OPA1 maintains proper OPA1 function and thereby maintains mitochondrial structural integrity (Samant et al., 2014). An additional Sirt3 deacetylase target is isocitrate dehydrogenase 2 (IDH2), a mitochondrial matrix protein that produces NADPH, a required substrate for several mitochondrial antioxidant enzymes. Sirt3 deacetylates IDH2, thereby maintaining IDH2 enzymatic activity and proper redox balance in the mitochondria (Someya et al., 2010; Yu et al., 2012). Mitochondrial ATP balance is maintained by Sirt3 deacetylation of ATP synthase β (ATPβ) (Zhang et al., 2016). Sirt3 aids in mitochondrial superoxide (O_2_•^−^) detoxification *via* deacetylation and activation of superoxide dismutase 2 (SOD2) (Qiu et al., 2010; Tao et al., 2014; Zhang et al., 2016; Dikalova et al., 2017). Increased electron transport chain flux can produce O_2_•^−^ due to premature transfer of electrons onto molecular oxygen from complex II (Nolfi-Donegan et al., 2020). Therefore, Sirt3 can fine-tune metabolic flux through the TCA cycle and electron transport chain, in tandem with mitigating the increased oxidant burden resulting from increased metabolic rates.

### Sirt5

Sirt5 is selective for negatively charged acyl moieties malonyl-, succinyl-, and glutaryl-lysine, but does not harbor detectable deacetylase activity (Table 1; Du et al., 2011; Lin et al., 2013; Park et al., 2013; Rardin et al., 2013; Tan et al., 2014; Nishida et al., 2015; Sadhukhan et al., 2016; Wang F. et al., 2017; Yang et al., 2017). Sirt5 is primarily localized to the mitochondria (Table 1; Michishita et al., 2005; Carrico et al., 2018); however, cytosolic localization of Sirt5 has also been demonstrated (Park et al., 2013). The deacylase activity of Sirt5 is important for regulating the activity of critical metabolic enzymes (Table 2). Sirt5 desuccinylation and deglutarylation activates carnitine palmitoyl synthase-1 (1), a urea cycle enzyme critical for the removal of ammonia from the cell (Du et al., 2011; Tan et al., 2014). Similarly, Sirt5 desuccinylates and activates serine hydroxymethyltransferase (SHMT2) (Yang et al., 2017), a mitochondrial enzyme important in purine biosynthesis, and activates ketogenesis through desuccinylation of mitochondrial hydroxymethylglutaryl-CoA synthase (HMGCS2) (Rardin et al., 2013). Sirt5 also desuccinylates and activates SOD1 (Lin et al., 2013), suggesting that both Sirt3 and Sirt5 play a joint role in regulating metabolism, as well as cellular oxidative stress burden.

### Sirt6

Unlike Sirt1 and Sirt2, which shuttle between the nucleus and cytosol, Sirt6 is restricted to the nucleus (Table 1; Michishita et al., 2005). Due to the presence of a hydrophobic tunnel in the Sirt6 acyl-lysine binding site, Sirt6 displays a preference for long-chain acyl groups, such as myristoyl-lysine (Table 1; Jiang et al., 2013; Feldman et al., 2015) and has almost undetectable deacetylase activity *in vitro* (Feldman et al., 2015). However, Sirt6 deacetylase activity is activated in the presence of fatty acids such as myristic acid (Feldman et al., 2013) and displays more robust deacetylase activity against whole histone substrates in cells (Table 2; Gil et al., 2013). Indeed, Sirt6 can deacetylate histone H3 at lysine 9 (H3K9) (Michishita et al., 2008), specifically at the NF-κB promoter, thereby repressing NF-κB-mediated transcriptional activation (Kawahara et al., 2009). Additionally, Sirt6 can deacetylate histone H3 at lysine 56 (H3K56) (Michishita et al., 2009). Sirt6 also promotes end resection at sites of DNA damage (Tian et al., 2019). Sirt6 can also serve as a mono-ADP-ribosyltransferase, both *in vitro* (Liszt et al., 2005) and *in vivo* (Mao Z. et al., 2011; Meter et al., 2014; Rezazadeh et al., 2019, 2020).

## Protein Oxidative Post-Translational Modifications

Sirtuins can be post-translationally modified and inhibited by many physiological oxidants including nitric oxide (NO), *S*-nitrosoglutathione (GSNO), hydrogen peroxide (H_2_O_2_), and peroxynitrite (ONOO^–^). The pathophysiological effect of oxidative stress at the whole organismal level is well established, and the importance of sirtuins in the molecular underpinnings of oxidative stress is becoming ever clearer (Merksamer et al., 2013; Santos et al., 2016; Shahgaldi and Kahmini, 2021). In chronic inflammatory disease states, the redox balance shifts to that of oxidative stress, where the normal metabolites of ROS and RNS increase disproportionately (Sies, 1997; Finkel and Holbrook, 2000; Jones, 2006). Of the amino acid side chains, cysteine and tyrosine are most susceptible to oxidation by ROS and RNS. The free thiol (or thiolate) of cysteine residues can react with NO-derived oxidants or nitrosonium donors such as GSNO (Smith and Marletta, 2012; Wynia-Smith and Smith, 2017; Massa et al., 2021), peroxides such as H_2_O_2_ (Yang et al., 2016) or ONOO^–^ (Alvarez and Radi, 2003), and glutathionylating agents such as glutathione disulfide (GSSG) or H_2_O_2_/glutathione (Kalinina and Novichkova, 2021). Overall, sirtuins are differentially sensitive to oxidative post-translational modification with distinct sets of oxidants. Here, we review the evidence for oxidative post-translational modification of each human sirtuin isoform, focusing on physiologically relevant oxidants and the resultant cysteine nitrosation [also referred to as nitrosylation (Smith and Marletta, 2012)], glutathionylation, sulfhydration, or sulfenylation or tyrosine nitration.

### Cysteine *S*-Nitrosation

Nitric oxide-derived oxidants can react with cysteine thiols, thiolates, or thiyl radicals to form *S*-nitrosothiols (RS-NO), which can occur *via* several mechanisms (Table 3; Smith and Marletta, 2012; Wynia-Smith and Smith, 2017; Massa et al., 2021). Free NO does not directly react with a thiol to generate a nitrosothiol and instead requires a one-electron oxidation, formally resulting in the net addition of NO^+^ to a cysteine thiolate (Smith and Marletta, 2012; Wynia-Smith and Smith, 2017). Under aerobic conditions, NO can react with molecular oxygen (O_2_) through several steps to eventually form N_2_O_3_ (Kharitonov et al., 1995; Keszler et al., 2010). A nucleophilic thiolate can then react with the electrophilic nitrogen of N_2_O_3_ to form a nitrosothiol and NO_2_^–^ (Keszler et al., 2010; Smith and Marletta, 2012; Wynia-Smith and Smith, 2017; Massa et al., 2021). NO can also directly add to a thiol to form a thionitroxyl radical (R-SNOH•) followed by a one electron oxidation to form a nitrosothiol in the presence of a protein-bound electron acceptor such as copper (II) and iron (III) (Smith and Marletta, 2012; Wynia-Smith and Smith, 2017). Nitrosothiols can also form *via* radical recombination between a cysteine thiyl radical and the unpaired electron of NO (Smith and Marletta, 2012; Wynia-Smith and Smith, 2017). Once nitrosothiols are formed, they can be transferred between proteins and small molecules, or between two proteins, *via* an S_*N*_2-like mechanism known as transnitrosation (Smith and Marletta, 2012; Wynia-Smith and Smith, 2017). A physiologically occurring small molecule that can transnitrosate proteins is GSNO (Broniowska et al., 2013). *S*-nitrosation is a reversible process; enzymes such as thioredoxin (Nikitovic and Holmgren, 1996; Stoyanovsky et al., 2005; Sengupta et al., 2007; Benhar et al., 2008) and GSNO reductase (Jensen et al., 1998; Liu et al., 2001) can restore nitrosated cysteines to their native sulfhydryl form.

**TABLE 3:** Oxidative modifications of human sirtuins, sites of modification, and associated references.

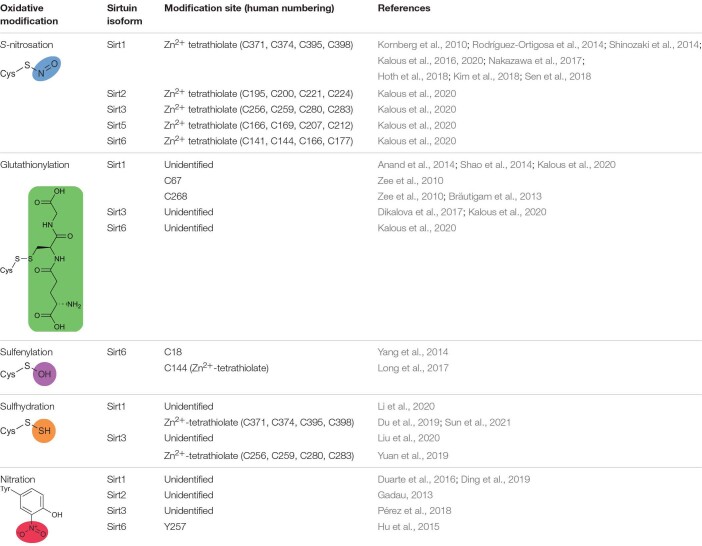

#### Sirt1 Is Modified and Inhibited by Zn^2+^-Tetrathiolate *S*-Nitrosation

A significant body of literature indicates that Sirt1 is both modified and inhibited by *S*-nitrosation (Table 3; Kornberg et al., 2010; Rodríguez-Ortigosa et al., 2014; Shinozaki et al., 2014; Kalous et al., 2016, 2020; Nakazawa et al., 2017; Hoth et al., 2018; Kim et al., 2018; Sen et al., 2018). Recombinantly purified Sirt1 shows direct transnitrosation *in vitro* following treatment with GSNO (Kornberg et al., 2010; Kalous et al., 2016, 2020; Goetz et al., 2020) or nitrosated GAPDH (Kornberg et al., 2010). Treatment with the transnitrosation donor *S*-nitroso-*N*-acetylpenicillamine (SNAP) (Shinozaki et al., 2014; Kalous et al., 2020) or NO-donating compounds (NONOates) (Kalous et al., 2016, 2020) also yields *S*-nitrosated Sirt1. A subsequent loss of Sirt1 deacetylase activity accompanies *S*-nitrosation (Kornberg et al., 2010; Shinozaki et al., 2014; Kalous et al., 2016, 2020). The *S*-nitrosation sites of Sirt1 were localized to Cys395 or Cys398 of the Zn^2+^-tetrathiolate *via* serine mutagenesis (Figures 1A,B; Shinozaki et al., 2014; Kalous et al., 2016). The structural integrity of the Zn^2+^-tetrathiolate is essential for Sirt1 deacetylase activity; treatment of recombinant Sirt1 with nitrosating agents or mutation of any of the four tetrathiolate cysteines to serine resulted in Zn^2+^ loss (Shinozaki et al., 2014; Kalous et al., 2016). A concomitant loss of secondary protein structure was seen *via* circular dichroism (Kalous et al., 2016). *S*-nitrosation of Sirt1 is reversible with thiol-based reductants (Shinozaki et al., 2014; Kalous et al., 2016); concurrent supplementation with free Zn^2+^ restores Sirt1 deacetylase activity *in vitro* (Kalous et al., 2016), implying restoration of the Zn^2+^-tetrathiolate motif secondary structure and that Sirt1 *S*-nitrosation may serve as a reversible signaling mechanism.

Sirt1 *S*-nitrosation and subsequent inhibition has been shown in multiple cultured cell lines and animal systems, and points to a close relationship with the activity of various nitric oxide synthase (NOS) isoforms. *S*-nitrosation of transfected Sirt1 was demonstrated in COS-7 cells in response to 300 μM SNAP or 600 μM GSNO treatment (Shinozaki et al., 2014). Disrupting the Zn^2+^-tetrathiolate by treating immunopurified Sirt1 from COS-7 cell lysates with the Zn^2+^ chelator TPEN resulted in inhibition of Sirt1 deacetylase activity (Shinozaki et al., 2014). *S*-nitrosation as an output of inflammation was further demonstrated in several cellular models, including C2C12 myotubes and Hepa1c1c7 hepatocytes treated with cytokines (Shinozaki et al., 2014). Moreover, *S*-nitrosation of Sirt1 was correlated with inhibition of Sirt1 deacetylase activity; increased Sirt1 *S*-nitrosation correlated with increased p53 and p65 acetylation, thereby activating apoptosis and promoting expression of proinflammatory genes (Shinozaki et al., 2014). Downstream effects of Sirt1 *S*-nitrosation were also investigated in cultured RAW 264.7 macrophages (Kim et al., 2018). Treatment of RAW 264.7 macrophages with 1 mM SNAP increased Sirt1 *S*-nitrosation, decreased Sirt1 deacetylase activity, and allowed HMGB1 release (Kim et al., 2018).

The primary source of NO in biological systems are the three NOS isoforms (Marletta, 1994). The two constitutively expressed NOS isoforms are the neuronal (nNOS or NOS1) and endothelial (eNOS or NOS3) isoforms (Knowles and Moncada, 1994). Both eNOS and nNOS activity respond to Ca^2+^ fluxes and are dependent on Ca^2+^/calmodulin-binding for activation (Stevens-Truss et al., 1997; Gribovskaja et al., 2005; Campbell et al., 2014). In HEK293 cells stably expressing nNOS, activation of nNOS with the Ca^2+^ ionophore A23187 induced Sirt1 *S*-nitrosation and increased PGC1α acetylation as a proxy for Sirt1 activity (Kornberg et al., 2010). The inducible NOS isoform (iNOS or NOS2) is induced in response to inflammatory inputs such as cytokines and lipopolysaccharide (LPS); iNOS activity increases in many inflammatory and age-related diseases (Anavi and Tirosh, 2019). In C2C12 myotubes and Hepa1c1c7 hepatic cells, pretreatment with cytokines induced iNOS expression, yielded *S*-nitrosated Sirt1, and increased p53 acetylation as a proxy for Sirt1 activity (Shinozaki et al., 2014).

Rodent models point to a whole organismal role of Sirt1 *S*-nitrosation. Primary hepatocytes from Wistar rats showed Sirt1 *S*-nitrosation when cultured in media supplemented with cholic acid to alter bile salt homeostasis (Rodríguez-Ortigosa et al., 2014). Furthermore, Sirt1 *S*-nitrosation was tied to iNOS and nNOS activity. In C57BL/6 mice injected with LPS, Sirt1 *S*-nitrosation was observed in the liver, along with increased acetylation and DNA binding of p53 and p65. These effects were not seen in iNOS knockout (iNOS^–/–^) animals (Shinozaki et al., 2014). A subsequent study examined Sirt1 *S*-nitrosation in C57BL/6 mice but with burn injury as the original inflammatory stimulus (Nakazawa et al., 2017). Sirt1 *S*-nitrosation seen following burn injury correlated with iNOS expression and increased p53 and p65 acetylation, implying Sirt1 inhibition (Nakazawa et al., 2017). Sirt1 *S*-nitrosation and inhibition was observed and tied to iNOS activity in a “two-hit” model of lung injury, wherein a pulmonary injury was induced in mice by artificial contusion, followed by either infection with the *Streptococcus pneumoniae* TIGR4 strain or exposure to LPS (Hoth et al., 2018). Inhibiting iNOS with the small molecule iNOS inhibitor 1400W (Garvey et al., 1997) prior to lung injury prevented Sirt1 inhibition as measured by a commercial fluorescent assay; however, the impact on protein targets of Sirt1 deacetylase activity was not assessed (Hoth et al., 2018). Relevance of this signaling cascade was also investigated systemically in the lungs of BALB/c mice treated with LPS; both increased Sirt1 *S*-nitrosation and extracellular HMGB1 were observed but inhibited by pretreatment with the iNOS inhibitor 1400W (Table 4; Kim et al., 2018). Moreover, in an aged rat model, *in vivo* Sirt1 *S*-nitrosation increased with age in an iNOS-dependent manner when comparing skeletal muscle of 2- and 28-month-old F344 rats (Shinozaki et al., 2014). Intraperitoneal injection of aged rats with the iNOS inhibitor 1400W reduced the level of Sirt1 *S*-nitrosation (Shinozaki et al., 2014).

**TABLE 4:** Known downstream *in vivo* effects of oxidative modifications of Sirt1 and Sirt3 in animal model systems.

**Sirtuin isoform**	**Oxidative modification**	**Downstream *in vivo* effects of oxidative modification**	**References**
Sirt1	*S*-nitrosation	Promotion of inflammatory gene activation in mouse neurons	Shinozaki et al., 2014
		Increased HMGB1 release in stimulated macrophages or injured mouse lung tissue	Kim et al., 2018
		Increased pathological tau acetylation in mouse cortical neurons	Sen et al., 2018
	Glutathionylation	Increased apoptosis in mice fed high fat diets	Shao et al., 2014
		Delayed and disordered zebrafish blood vessel network formation	Bräutigam et al., 2013
	Nitration	In high glucose conditions, increased acetylated p65 in diabetic mouse retina	Duarte et al., 2016
		Nicotine-induced decreased Sirt1 activity and downstream YAP activation leading to mouse aorta arterial stiffness	Ding et al., 2019
Sirt3	Glutathionylation	Reduced SOD2 activity in mouse and human hypertension	Dikalova et al., 2017

Sirt1 *S*-nitrosation was also demonstrated in neurodegenerative models. In Parkinson’s disease, excess NO may be constitutively present due to increased nNOS activity (Hantraye et al., 1996; Przedborski et al., 1996). In an induced Parkinson’s disease mouse model, increased Sirt1 *S*-nitrosation paralleled increased p53 and p65 acetylation, and Sirt1 *S*-nitrosation was reduced when nNOS was inhibited with 7-nitroindazole (Table 4; Shinozaki et al., 2014). In a mouse model of Alzheimer’s disease, nNOS was also tied to Sirt1 *S*-nitrosation. Isolated primary cortical neurons of C57BL/6 mice were exposed to aggregated amyloid-β 1–42 peptide (Aβ_1__–__42_) (Sen et al., 2018). Aβ_1__–__42_ is thought to be the pathogenic form that accumulates and induces toxicity within neurons in Alzheimer’s disease. Neuronal exposure to Aβ_1__–__42_ induced *S*-nitrosation of GAPDH and Sirt1, decreased the Sirt1-tau interaction, and increased tau acetylation in an nNOS-dependent manner (Table 4; Sen et al., 2018).

Together, these animal models suggest Sirt1 *S*-nitrosation occurs *in vivo* under conditions of inflammation, aging, and neurodegeneration. Pharmacological prevention or reversal of Sirt1 *S*-nitrosation resulting in a net increase in Sirt1 deacetylase activity may be beneficial in these disease contexts.

#### Sirt2 Demyristoylase Activity Is Inhibited by Zn^2+^-Tetrathiolate *S*-Nitrosation *in vitro*

Recombinantly purified Sirt2, like its nuclear/cytosolic counterpart Sirt1, can be inhibited by *S*-nitrosation *in vitro* (Kalous et al., 2020; Table 3). A concentration-dependent increase in Sirt2 *S*-nitrosation was observed by treatment with GSNO and NO (up to 100 μM), correlating with inhibition of Sirt2 demyristoylase activity. As demonstrated by the ratio of the maximum rate of inactivation to the apparent covalent inhibition constant (*k*_inact_/*K*_I_), Sirt2 was the most efficiently inactivated by GSNO compared to Sirt1 and Sirt6, suggesting Sirt2 is more likely to be inactivated by GSNO compared to Sirt1 or Sirt6 in a cellular context (Kalous et al., 2020). Mutagenesis of the four Zn^2+^-tetrathiolate cysteines to alanine abolished *S*-nitrosation, indicating the Zn^2+^-tetrathiolate is the likely site of *S*-nitrosation (Kalous et al., 2020). Future studies are needed to determine if Sirt2 is modified and inhibited by *S*-nitrosation in cellular or animal models.

#### Sirt3 and Sirt5 Are Modified but Not Inhibited by Zn^2+^-Tetrathiolate *S*-Nitrosation *in vitro*

*In vitro* exposure of Sirt3 to NO (100 μM released from a NONOate), but not 100 μM GSNO, resulted in cysteine *S*-nitrosation (Table 3; Kalous et al., 2020). However, Sirt3 deacetylase activity was not significantly inhibited by NO. Similarly, the *in vitro* sensitivity of Sirt5 to NO-derived oxidants demonstrated Sirt5 *S*-nitrosation, but Sirt5 desuccinylase activity was not inhibited (Kalous et al., 2020). As discussed in section “Sirt1 Can Be Modified and Inhibited by Glutathionylation,” Sirt1 *S*-nitrosation occurs selectively at the Zn^2+^-tetrathiolate leading to inhibition of Sirt1 deacetylase activity (Shinozaki et al., 2014; Kalous et al., 2016). Likewise, mutation of the Zn^2+^-tetrathiolate cysteine residues to alanine abolished *S*-nitrosation signal in Sirt3 and Sirt5 (Kalous et al., 2020), but failed to inhibit deacylase activity. This decoupling of Sirt3 and Sirt5 Zn^2+^-tetrathiolate *S*-nitrosation and inhibition suggests that instead of directly inhibiting deacetylase activity, *S*-nitrosation may serve as a degradation signal similar to effects of Sirt3 glutathionylation (discussed in section “Sirt3 Can Be Modified and Inhibited by Glutathionylation”). Alternatively, the Sirt3 or Sirt5 Zn^2+^-tetrathiolate may be modified, but the integrity of the Zn^2+^-tetrathiolate may not be critical for Sirt3 or Sirt5 deacylase activity or disruption of the Zn^2+^-tetrathiolate may only alter binding and deacetylation of specific protein substrates *in vivo*, an effect missed when using acetylated peptide substrates *in vitro*. Alternatively, Sirt3 and Sirt5 may have evolved insensitivity to oxidants to maintain the deacylase activity of these mitochondrially targeted isoforms, as the mitochondrial environment is a primary source of ROS and RNS.

#### Sirt6 Is Inhibited by Zn^2+^-Tetrathiolate *S*-Nitrosation *in vitro*

In our *in vitro* NO-derived oxidant screen, Sirt6 displayed similar patterns of oxidative modification and inhibition to the other nuclear sirtuin isoforms Sirt1 and Sirt2. Sirt6 was *S*-nitrosated and demyristoylase activity inhibited in a concentration-dependent manner by NO and nitrosothiols such as GSNO (Table 3; Kalous et al., 2020). Mutagenesis of Zn^2+^-tetrathiolate cysteines to alanine indicated the Sirt6 Zn^2+^-tetrathiolate is the likely site of modification (Figures 1A,C; Kalous et al., 2020). Of the sirtuins examined in our study (Sirt1, Sirt2, Sirt3, Sirt5, and Sirt6), Sirt2 and Sirt6 were the only isoforms sensitive to inhibition by NO (released from a NONOate), where the second-order rate constant for Sirt6 inactivation measured by *k*_inact_/*K*_I_ analysis was similar to that of Sirt2, suggesting that Sirt6 and Sirt2 are the most likely sirtuins to be inactivated by reaction with NO-derived oxidants compared to the other human sirtuins (Kalous et al., 2020). Future studies are needed to determine if Sirt6 is modified and inhibited by *S*-nitrosation in cellular or animal models.

### Cysteine Glutathionylation

The thiol of cysteine residues can be covalently and reversibly modified by glutathione to yield glutathionylated (RS-SG) protein (Table 3). Multiple pathways lead to protein glutathionylation. Formation of nitrosothiols or sulfenic acids on a protein or GSH can precede glutathionylation, whereby GSH reacts with sulfenylated or nitrosated cysteines to yield glutathionylated protein and H_2_O or HNO, respectively. For example, in the presence of H_2_O_2_ and reduced glutathione (GSH), H_2_O_2_ can react with a protein cysteine thiol to form a transient sulfenic acid, followed by nucleophilic attack of the sulfenic acid by the GSH thiol resulting in glutathionylation (Kalinina and Novichkova, 2021). Alternatively, in a thiol-disulfide exchange reaction, GSSG can react with a cysteine thiol to yield a glutathionylated cysteine and GSH. Glutathionylation can also be catalyzed by glutaredoxin (Grx), where a thiyl radical (RS•) interacts with glutathione thiyl radical (GS•) (Ghezzi, 2013). Like other oxidative modifications, addition of the glutathione moiety on a reactive cysteine can perturb the global structure and activity of a protein, often inhibiting activity (Ghezzi, 2013). Glutathionylation is reversible, with glutaredoxin enzymes catalyzing the removal of the modification (Jung and Thomas, 1996; Chrestensen et al., 2000).

#### Sirt1 Can Be Modified and Inhibited by Glutathionylation

In addition to low micromolar GSNO treatment (6–100 μM) yielding Sirt1 *S*-nitrosation and inhibition of deacetylase activity (Kalous et al., 2016, 2020), Sirt1 can also be glutathionylated upon high concentration (2 mM) GSNO exposure (Table 3; Zee et al., 2010). It is important to note that GSNO preparations can be contaminated with significant amounts of GSSG (Becker et al., 1995; Ji et al., 1999; Giustarini et al., 2005), such that glutathionylation may result from reaction with GSSG instead of GSNO especially when treating with high GSNO concentrations. Mass spectrometry of FLAG-purified Sirt1 treated with 2 mM GSNO from HEK293 or bovine aortic endothelial cell (BAEC) lysate identified Cys67, Cys268, Cys326, and Cys623 as potential glutathionylated residues (Zee et al., 2010). However, instead of using a nitrosothiol-selective reductant such as ascorbate to differentiate the relative contribution of GSNO to *S*-nitrosation versus glutathionylation, DTT was the only reductant used, which reduces both modifications. Indeed, although five sites of GSNO-dependent modification were initially detected, only one, Cys67 (Figures 1A,B), was definitively identified as glutathionylated upon treatment with 2 mM GSNO (Zee et al., 2010). Therefore, the other sites identified may be sites of *S*-nitrosation or other GSNO-dependent redox-dependent modification such as inter- or intra-molecular disulfide formation.

While both *S*-nitrosation and glutathionylation are possible outcomes of a reaction between GSNO and a protein, *S*-nitrosation is the more kinetically favorable reaction (Gallogly and Mieyal, 2007). To deconvolute the relative ability of GSNO to nitrosate versus glutathionylate Sirt1, recombinantly purified Sirt1 was incubated with increasing concentrations of GSNO and trends in *S*-nitrosation and glutathionylation were observed (Kalous et al., 2016). Sirt1 was both glutathionylated and *S*-nitrosated by GSNO; however, the concentration dependence was vastly different. While Sirt1 *S*-nitrosation occurred in a concentration-dependent manner that directly paralleled Sirt1 inhibition and began at ∼6 μM GSNO, Sirt1 glutathionylation was not observed until 100 μM GSNO, consistent with *S*-nitrosation being the more favorable reaction. Both *S*-nitrosation and glutathionylation may collectively contribute to Sirt1 inhibition at concentrations higher than 100 μM. However, as GSNO and other *S*-nitrosothiols likely to accumulate only to nanomolar to low micromolar concentrations in cells (Broniowska and Hogg, 2012; Seneviratne et al., 2013), *S*-nitrosation is likely the physiologically relevant Sirt1 modification.

The deacetylase activity of recombinant zebrafish Sirt1 treated with supraphysiological GSSG concentrations (5 mM) showed 17% the deacetylase activity of untreated Sirt1 (Bräutigam et al., 2013). Incubation of glutathionylated Sirt1 with a fivefold molar excess of the oxidoreductase enzyme glutaredoxin-2 (Grx2) removed nearly all glutathione moieties and restored enzymatic activity to 63% that of fully reduced Sirt1, suggesting glutathionylation is a reversible process. A primary site of Sirt1 glutathionylation was localized to Cys204 (corresponds to Cys268 in the human isoform; Figures 1A,B); the cysteine to serine mutant significantly reduced the glutathionylation signal and deacetylase activity but did not completely abolish either. Furthermore, incubation of glutathionylated Sirt1 with Grx2 did not fully deglutathionylate Sirt1 or fully restore deacetylase activity. Taken together, this suggests Sirt1 can be glutathionylated at more than one site.

Cell-based studies suggest glutathionylation can inhibit Sirt1 activity (Shao et al., 2014). In HepG2 cells under conditions mimicking metabolic stress (high palmitate and high glucose) or treatment with *S*-nitrosocysteine (CysNO), Sirt1 glutathionylation was demonstrated by immunoblot (Shao et al., 2014). Overexpression of the deglutathionylating enzyme glutaredoxin-1 (Grx1) maintained endogenous Sirt1 activity and prevented apoptotic signaling in metabolically stressed HepG2 cells. Mutating a subset of potential glutathionylation target residues initially identified by Zee et al. (2010) abolished glutathionylation in a Sirt1 triple Cys-to-Ser mutant (Cys67, Cys326, and Cys623) (Shao et al., 2014). The oxidation-resistant Sirt1 triple mutant was further used in a study examining a potential glutathionylation-dependent interaction between GAPDH and Sirt1 (Rizvi et al., 2021). Though a definitive site of modification on Sirt1 was not identified, the triple mutant reduced glutathionylation signal and interaction with glutathionylated GAPDH *via* immunoblot (Rizvi et al., 2021). It was surprising that an established transnitrosating agent such as CysNO increased glutathione adducts on Sirt1 (Shao et al., 2014). However, *S*-nitrosation may serve as a precursor to glutathionylation by GSH. Importantly, these glutathionylation assays were performed using DTT (Shao et al., 2014) or TCEP (Rizvi et al., 2021) as a reductant prior to biotin labeling of putative glutathionylated cysteines rather than employing a nitrosothiol-selective reductant such as ascorbate. This confounds specificity in the modification identified; DTT and TCEP reduce both nitrosothiols and disulfides (and the other cysteine modifications discussed herein), indicating that treatment with CysNO may in fact result in *S*-nitrosation.

In animal models, Sirt1 glutathionylation occurs during oxidative stress. Sirt1 glutathionylation was observed in livers of C57/B6J mice fed a high fat, high-sucrose diet, which promoted production of ROS (assessed *via* dichlorodihydrofluorescein fluorescence), inhibition of Sirt1 deacetylase activity, and apoptosis *via* increased p53 acetylation (Table 4; Shao et al., 2014). A similar phenomenon was demonstrated in zebrafish, where Sirt1 glutathionylation correlated with delayed and disordered blood vessel network formation (Table 4; Bräutigam et al., 2013). Glutathionylated Sirt1 was observed in lungs of C57BL/6J mice exposed to cigarette smoke (Caito et al., 2010). Inhaled cigarette smoke increased Sirt1 glutathionylation, which was further augmented in Grx1 knockout (Grx1^–/–^) mice (Caito et al., 2010). Additionally, Grx1 deletion alone increased Sirt1 glutathionylation, even in the absence of cigarette smoke exposure (Caito et al., 2010).

#### Sirt3 Can Be Modified and Inhibited by Glutathionylation

Sirt3 glutathionylation has been observed in response to direct exposure of recombinantly purified Sirt3 to glutathionylating agents and in response to physiological inflammatory stimuli (Table 3; Dikalova et al., 2017; Kalous et al., 2020). *In vitro* exposure of recombinantly purified Sirt3 to 100 μM H_2_O_2_/GSH resulted in glutathionylation by anti-glutathione but no change in Sirt3 deacetylase activity (Kalous et al., 2020). Sirt3 is more sensitive to glutathionylation by 100 μM H_2_O_2_/GSH than by 100 μM GSSG, with significantly higher anti-glutathione signal observed in response to 100 μM H_2_O_2_/GSH (Dikalova et al., 2017; Kalous et al., 2020).

Sirt3 glutathionylation has been observed in animal models. Sirt3 is glutathionylated *in vivo* in the aorta and kidney of hypertensive mice, which correlated with increased acetylation of the Sirt3 substrate SOD2, suggesting Sirt3 deacetylase activity is inhibited *in vivo* under inflammatory conditions (Dikalova et al., 2017). In clinically hypertensive human subjects, although Sirt3 glutathionylation was not examined, SOD2 acetylation was significantly increased, suggesting that both Sirt3 deacetylase and SOD2 dismutase activity are inhibited in hypertension (Table 4; Dikalova et al., 2017). Although increased Sirt3 glutathionylation was observed cellularly, Sirt3 protein levels were also reduced by ∼50%, suggesting that, instead of directly inhibiting Sirt3 activity, glutathionylation may destabilize Sirt3 or serve as a degradation signal *in vivo*, resulting in loss of Sirt3 activity *via* depletion of Sirt3 protein levels (Dikalova et al., 2017). Taken together, Sirt3 glutathionylation may either promote degradation of Sirt3, alter the Sirt3 interactome, or as a direct inhibitory modification under inflammatory conditions.

#### Sirt5 Is Not Glutathionylated *in vitro*

Sirt5 has only been examined *in vitro* to assess glutathionylation status. Recombinantly purified Sirt5 was not glutathionylated by treatment with conditions (100 μM GSNO, GSSG, or H_2_O_2_/GSH) that yielded glutathionylated Sirt3 (Table 3; Kalous et al., 2020).

#### Sirt6 Glutathionylation *in vitro* Does Not Inhibit Demyristoylase Activity

A single study investigated the glutathionylation state of recombinantly purified Sirt6. Anti-glutathione immunoblots showed Sirt6 glutathionylation when treated with either GSSG or H_2_O_2_/GSH (100 μM) (Table 3; Kalous et al., 2020). However, purified Sirt6 demyristoylase activity was not significantly inhibited by 100 μM GSSG treatment, suggesting glutathionylation is not an inhibitory modification.

### Cysteine Sulfenylation

Sulfenylation is a reversible oxidative modification where cysteine thiol is converted to a sulfenic acid (RS-OH) by reaction with two electron oxidants such as the peroxides H_2_O_2_ or ONOO^–^ (Table 3; Alvarez and Radi, 2003; Yang et al., 2016); cysteine sulfenylation is reducible by common intracellular reductants such as reduced glutathione (Reddie and Carroll, 2008; Conte and Carroll, 2013; Gupta and Carroll, 2014). In recent years, protein sulfenylation has been described in numerous systems due to the advent of dimedone-based and other sulfenylation-selective probes (Holmila et al., 2018; Shi and Carroll, 2020, 2021). In *in vitro* studies of recombinantly purified Sirt1, Sirt2, Sirt3, and Sirt5, sulfenylation was not detected by the DYn-2 dimedone-based probe following treatment with 100 μM H_2_O_2_ (Kalous et al., 2016, 2020). Sirt6 is thus unique among sirtuins in that Sirt6 is the only isoform identified as sulfenylated (Yang et al., 2014; Long et al., 2017). Sirt6 contains five cysteine residues, four of which are conserved residues comprising the Zn^2+^-tetrathiolate.

#### Sirt6 Can Be Sulfenylated in Cells Leading to Disulfide Formation or Inhibition

In a recent study, RKO colon adenocarcinoma cells were treated with 500 μM H_2_O_2_, and A4331 epidermal carcinoma cells were stimulated with EGF to mimic inflammatory conditions. In both cases, robust Sirt6 sulfenylation was observed exclusively at a cysteine residue unique to Sirt6 (Cys18; Figures 1A,C) *via* DYn-2 probe followed by LC-MS/MS analysis (Table 3; Yang et al., 2014). Incubation of recombinantly purified Sirt6 and hypoxia-inducible factor 1-α (HIF1α) with 500 μM H_2_O_2_ demonstrated electromobility shifts in non-reducing gels for both Sirt6 and HIF1α, consistent with formation of a Sirt6-HIF1α dimer (Yang et al., 2014). Dimerization was mediated by a disulfide and dependent on cysteine sulfenylation, as the Sirt6-HIF1α dimer was not observed in the absence of H_2_O_2_, or in the presence of H_2_O_2_ under reducing conditions. Moreover, formation of a Sirt6-HIF1α dimer was prevented by cotreatment with 500 μM H_2_O_2_ and 10 mM dimedone, which selectively and covalently labels sites of cysteine sulfenylation. Mass spectrometry identified an intermolecular disulfide between Sirt6 Cys18 and HIF1α Cys800. An important caveat to these results is that this study employed a supraphysiological H_2_O_2_ concentration (500 μM) (Yang et al., 2014). Physiological H_2_O_2_ concentrations are in the low micromolar range at maximum (Sies and Jones, 2020). Indeed, a more conservative treatment of recombinantly purified Sirt6 with 100 μM H_2_O_2_ showed no sulfenylation (Kalous et al., 2020).

Sirt6 sulfenylation was also detected in THP-1 monocytes stimulated with LPS; however, this study localized the sulfenic acid to Cys144 (Figures 1A,C), one of the four Sirt6 Zn^2+^-tetrathiolate cysteines (Long et al., 2017). Sulfenylation correlated with reduced Sirt6 deacetylase activity and increased GLUT1 translocation to the cell membrane and increased glycolytic activity in THP1 monocytes (Long et al., 2017). Interestingly, HIF1α regulates GLUT1 transcription (Chen et al., 2001). These data therefore provide physiological evidence that Sirt6 plays a repressive role in modulating HIF1α signaling.

### Cysteine Sulfhydration

Sulfhydration (also referred to as persulfidation or sulfuration) is a posttranslational modification where a cysteine sulfhydryl group is modified to the persulfide form (RS-SH) (Table 3). This modification may arise through several mechanisms. First, a cysteine side chain may first undergo oxidation (e.g., sulfenylation or glutathionylation), followed by reaction with H_2_S or HS^–^ to yield a persulfide bond (Paul and Snyder, 2012). Once formed, persulfides have been speculated to trans-*S*-sulfhydrate neighboring cysteine residues (Zhang et al., 2017) similar to transnitrosation (section “Cysteine *S*-Nitrosation”). A sulfhydrated cysteine is more reactive than the native thiol due to the lower p*K*_*a*_ value of a sulfhydrated cysteine (CysSSH p*K*_*a*_ = 4.3; CysSH p*K*_*a*_ = 8.3) (Cuevasanta et al., 2015; Shi and Carroll, 2020), which has downstream potential implications for protein structure, interacting proteins, and signaling pathways (Paul and Snyder, 2012).

#### Sirt1 Can Be Modified and Activated by Zn^2+^-Tetrathiolate Sulfhydration in Cells

Sirt1 sulfhydration has been demonstrated in cellular systems. In a 1-methyl-4-phenylpyridinium (MPP+) induced neurotoxicity model, SH-SY5Y neuroblastoma cells treated with 500 μM of the H_2_S donor NaHS prevented morphological and apoptotic effects of MPP+, and a modified biotin switch technique revealed increased sulfhydration of Sirt1 (Li et al., 2020). In the hepatic HepG2 cell line, increasing the output of H_2_S-producing enzyme cystathionine gamma-lyase (CSE) or treatment with NaHS led to an increase in both Sirt1 expression levels and deacetylase activity (Du et al., 2019). A complementary study in the human kidney HK-2 cell line found treatment with the polysulfide donor Na_2_S_4_ reversed effects of high glucose treatment; glucose-induced reduction in Sirt1 protein level was reversed, along with reversal of glucose-induced increases in STAT3 and p65 acetylation (Sun et al., 2021). Sirt1 sulfhydration was detected by Western blot following modified biotin switch; mutagenesis identified the modification site as the Zn^2+^-tetrathiolate (Du et al., 2019; Sun et al., 2021).

#### Sirt3 Can Be Modified and Activated by Zn^2+^-Tetrathiolate Sulfhydration in Cells

Sirt3 sulfhydration has been reported in multiple systems. In a model of chemotherapy-induced toxicity, human kidney cells (HK-2) or AKI mice were treated with cisplatin; basal levels of Sirt3 sulfhydration decreased, but rebounded upon NaHS treatment (Yuan et al., 2019). Likewise, in a liver toxicity model, the rat liver cell line BRL-3 was treated with the pesticide paraquat, or paraquat in combination with NaHS; paraquat treatment reduced sulfhydrated Sirt3 whereas co-treatment with NaHS restored Sirt3 sulfhydration (Liu et al., 2020). Functional downstream effects of Sirt3 sulfhydration in HK-2 cells included activation of Sirt3-catalyzed deacetylation of OPA1, SOD2, and ATP synthase subunit β (Yuan et al., 2019). The Zn^2+^-tetrathiolate was identified as the modification site *via* mutagenesis (Yuan et al., 2019).

### Tyrosine Nitration

Tyrosine nitration is a post-translational modification whereby a nitro group (−NO_2_) is covalently substituted for a hydrogen in the three position ortho to the tyrosine phenolic sidechain hydroxyl (Table 3; Campolo et al., 2020). The first step in formation of 3-nitrotyrosine is oxidation of the tyrosine sidechain hydroxyl to the tyrosyl radical (Tyr•). Conversion of the tyrosyl radical to 3-nitrotyrosine can occur by reaction with ONOO^–^, NO radical, radical nitrate (•NO_2_), or by an enzymatic process involving myeloperoxidase, NO_2_^–^, and H_2_O_2_ (Bartesaghi and Radi, 2018). Like other oxidative modifications discussed in this review, tyrosine nitration is implicated in numerous inflammatory conditions, including cancer, neurodegeneration, and cardiovascular disease (Bartesaghi and Radi, 2018).

#### Sirt1 Can Be Modified and Inhibited by Tyrosine Nitration

Although the precise site(s) of nitration were not identified, global Sirt1 tyrosine nitration was demonstrated in both the retinas of mice and rats with diabetic retinopathy *via* microscopy (Table 3; Duarte et al., 2016). Under high glucose conditions, Sirt1 deacetylase activity decreased, as measured *via* an *in vitro* fluorometric assay and an increase in p65 acetylation. Direct detection of tyrosine nitration of immunopurified Sirt1 was visualized by immunoblot. Additionally, silencing NADPH oxidase 4 (NOX4) under high glucose conditions prevented Sirt1 inhibition, suggesting NOX4 is the primary source of O_2_•^−^ production responsible for ONOO^–^ formation (*via* radical recombination between O_2_•^−^ and NO) and Sirt1 inhibition under these conditions (Table 4; Duarte et al., 2016). In mouse aorta treated with nicotine, a microscopic overlay of Sirt1 and anti-3-nitrotyrosine showed co-localization (Table 4; Ding et al., 2019). Sirt1 deacetylase activity was also decreased, and Zn^2+^ was released from recombinant Sirt1 upon 50 μM OONO^–^ treatment, indicating tyrosine nitration of Sirt1 may be an inhibitory modification (Ding et al., 2019).

#### Sirt2 Inhibition Correlates With Increased Global Tyrosine Nitration

Sirt2 tyrosine nitration was indirectly implicated in rat glia Schwannoma cells treated with high glucose. An increase of global nitration correlated with increased acetylated α-tubulin and decreased Sirt2 protein (Table 3; Gadau, 2013), consistent with Sirt2 inhibition. Acetylated α-tubulin also correlated with dysregulation of the microtubular network (Gadau, 2013), which represents a potential pathway through which the structural changes observed in diabetic neuropathy occur. Future studies are needed to determine if Sirt2 nitration is directly responsible for the observed increased α-tubulin acetylation in Schwannoma cells.

#### Sirt3 Can Undergo Nitric Oxide Synthase-Dependent Tyrosine Nitration

Recombinantly purified Sirt3 treated with 100–500 μM ONOO^–^ showed a concentration-dependent inhibition by an *in vitro* fluorometric assay (Table 3; Pérez et al., 2018). Increased Sirt3 nitration in mouse brain of aged (24-month-old) relative to young (3-month-old) animals has also been observed. The source of nitration was nNOS-dependent and activated by ROS-regulating adaptor protein p66^Shc^, as whole-body deletion of p66^Shc^ resulted in significantly reduced mitochondrial protein nitration in aged p66^Shc^ knockout (p66^Shc–/–^) mice. p66^*S*hc^ can activate NAPDH oxidase, thereby increasing the O_2_•^–^ production (Pérez et al., 2018) necessary for ONOO^–^ formation. If sufficient ONOO^–^ is produced to inhibit Sirt3, increased acetylation and decreased activity of critical antioxidant and other mitochondrial metabolic enzymes may result, which requires further study.

#### Sirt6 Can Be Modified and Inhibited by Tyrosine Nitration

Recombinantly purified Sirt6 showed tyrosine nitration and deacetylase inhibition when treated with the OONO^–^ donor SIN-1 at high concentrations (0.5 and 5 mM) (Table 3); the specific site of modification was identified as Tyr257 by mass spectrometry analysis (Figures 1A,C; Hu et al., 2015). Sirt6 tyrosine nitration and deacylase inhibition, as determined by a fluorometric assay, was likewise demonstrated in HEK293 cells and human retinal microvascular endothelial cells treated with 0.5 or 5 mM of SIN-1 (Hu et al., 2015). Nitration and inhibition of Sirt6 was also observed in the retina of mice where systemic inflammation was induced by injection of LPS, suggesting nitration may be a relevant inhibitory modification of Sirt6 *in vivo* (Hu et al., 2015). Oxidants such as NO and ONOO^–^ cause both single and double-stranded breaks in DNA (Burney et al., 1999), and Sirt6 facilitates DNA damage repair to increase the efficiency of strand break repair (Tian et al., 2019). However, if DNA damage is too extensive, protective mechanisms are shut down, and the cell is directed toward apoptosis (Roos and Kaina, 2013). In this case, Sirt6 inhibition by oxidants may serve as a mechanism to inhibit pro-survival responses once oxidant-dependent DNA damage becomes too great to repair.

## Conclusion

In summary, sirtuins are susceptible to oxidative post-translational modifications such as cysteine nitrosation, glutathionylation, sulfhydration, and sulfenylation as well as tyrosine nitration in an isoform-specific manner. A limitation of many studies is the use of supraphysiological concentrations of oxidants. While oxidative post-translational modifications of sirtuins have been detected, whether the modification itself is causative of sirtuin inhibition or correlative with inhibition of sirtuins *via* depletion of NAD^+^ or modulating other regulatory post-translational modification (e.g., phosphorylation) or protein–protein interactions is unclear, as NAD^+^ levels and other post-translational modifications and protein–protein interactions were not measured in any study examining sirtuin oxidation. The capacity of sirtuins to be post-translationally modified in a cellular context in response to exogenous inflammatory stimuli has been demonstrated; further work to identify endogenous factors is still needed. Understanding of the inflammatory stimuli acting to inhibit sirtuin activity in aging-related diseases will help pave the way to developing therapeutics targeted at modulated these oxidative post-translational modifications of sirtuins.

## Author Contributions

KK and SW-S wrote the manuscript. SW-S and BS reviewed and edited the manuscript. All authors contributed to the article and approved the submitted version.

## Conflict of Interest

The authors declare that the research was conducted in the absence of any commercial or financial relationships that could be construed as a potential conflict of interest.

## Publisher’s Note

All claims expressed in this article are solely those of the authors and do not necessarily represent those of their affiliated organizations, or those of the publisher, the editors and the reviewers. Any product that may be evaluated in this article, or claim that may be made by its manufacturer, is not guaranteed or endorsed by the publisher.
